# Nanostructured Materials for Artificial Tissue Replacements

**DOI:** 10.3390/ijms21072521

**Published:** 2020-04-05

**Authors:** Jana Pryjmaková, Markéta Kaimlová, Tomáš Hubáček, Václav Švorčík, Jakub Siegel

**Affiliations:** 1 Department of Solid State Engineering, University of Chemistry and Technology Prague, Technická 5, 166 28 Prague, Czech Republic; jana.pryjmakova@vscht.cz (J.P.); marketa.kaimlova@vscht.cz (M.K.); vaclav.svorcik@vscht.cz (V.Š.); 2 Soil & Water Research Infrastructure, Biology Centre CAS, Na Sádkách 7, 370 05 České Budějovice, Czech Republic; tomas.hubacek@hbu.cas.cz

**Keywords:** nanomaterials, tissue engineering, biologic properties, mechanical properties, antibacterial effects

## Abstract

This paper review current trends in applications of nanomaterials in tissue engineering. Nanomaterials applicable in this area can be divided into two groups: organic and inorganic. Organic nanomaterials are especially used for the preparation of highly porous scaffolds for cell cultivation and are represented by polymeric nanofibers. Inorganic nanomaterials are implemented as they stand or dispersed in matrices promoting their functional properties while preserving high level of biocompatibility. They are used in various forms (e.g., nano- particles, -tubes and -fibers)—and when forming the composites with organic matrices—are able to enhance many resulting properties (biologic, mechanical, electrical and/or antibacterial). For this reason, this contribution points especially to such type of composite nanomaterials. Basic information on classification, properties and application potential of single nanostructures, as well as complex scaffolds suitable for 3D tissues reconstruction is provided. Examples of practical usage of these structures are demonstrated on cartilage, bone, neural, cardiac and skin tissue regeneration and replacements. Nanomaterials open up new ways of treatments in almost all areas of current tissue regeneration, especially in tissue support or cell proliferation and growth. They significantly promote tissue rebuilding by direct replacement of damaged tissues.

## 1. Introduction

Nanomaterials, exceptional form of matter with dimensions as small as one billionth of meter, are commonly defined with dimension range 1–100 nm. They may have different shapes and complex morphologies which enable to classify them into three basic groups, i.e., 0, 1 and 2 dimensional (0, 1 and 2D) nanomaterials as they are smaller than 100 nm in all directions, two axes or one axis, respectively. Nanomaterials have many shapes, whereas the most common ones are nanoparticles (0D) [1], nanowires (1D) [2], nanolayers (2D) [3]. All together, they represent basic building blocks or active components in dynamically growing field of bioengineering.

Owing to their nanoscale dimension, nanomaterials possess properties resulting from critical physical and chemical characteristics. This predetermines such materials for a wide range of utility in biologic systems. Nanomaterials are used in tissue engineering [4], drug delivery [5], bioimaging [6], gene therapy [7], etc. The other desired property is their large surface to volume ratio. It makes nanoparticles (NPs) possible to go through the membrane and help cells absorb proteins [8,9,10]. For this purpose, lipid [8], chitosan [9] and inorganic [10] (e.g., calcium phosphate, gold, carbon, silicon oxide and iron oxide) NPs have already been used. In that way, NPs can imitate the natural nanometer-size scale extracellular matrix components and enables direct delivery of biologically active agents. Another reason for the usage of different forms of nanomaterials in biologic applications is the alteration of properties to required ones in tissue engineering.

Nanostructured materials may be of various origin and chemical composition organic and inorganic ones. In tissue engineering, the organic nanomaterials often include polymeric nanofibers, which may serve as a scaffold for cell cultivation [11]. More opportunities for such purposes represent various inorganic nanomaterials [12]. By their incorporation into organic matrix scaffolds better mimicking of physiological environment can be achieved. The resulting composites can show unique properties required for assumed applications in hard and soft tissue engineering [13].

The aim of tissue engineering is to prepare biologically equivalent tissue or organ replacements. The construction of the cell carriers (scaffold) is based on the knowledge of nanotechnology, but the subsequent colonization of these carriers by the required population of cells is based on the knowledge of cell and molecular biology and tissue physiology. So that, it is a multidisciplinary field that requires understanding of both material and biochemical aspects important for prevention of undesirable immune response of acceptor site of implant [14]. Thus, the material of the scaffold must be carefully chosen, and for better cell conformity, the material properties properly modified [15]. Nowadays, there are lot of well described nanostructured organic/inorganic composite materials for effective reparation or replacement of portions or whole tissues (i.e., cartilage [16], bone [17], neural [18], cardiac [19] and skin [20] tissue replacement etc.), where inorganic materials play irreplaceable role in the properties modification. This review is predominantly focused on this type of composites.

## 2. Organic Nanomaterials

Organic materials applicable in tissue engineering are predominantly used as scaffolds for a cell cultivation. The prevailing group of scaffold materials are polymers, especially polymeric nanofibers [11]. Polymeric nanofiber networks are able to mimic the constitution of physiological human tissue at the nanometer scale. The networks support cell adhesion, proliferation, migration and differentiation with high efficacy, because they exhibit high surface-to-volume ratio [21]. Compared with microfiber-based scaffolds, they excel in greater mechanical strength [22]. Nanofiber properties depend on their orientation. Randomly oriented nanofibers increase material stiffness and mechanical resistance in all directions. Contrary to that, arranged (so-called aligned) nanofibers enhance mechanical properties only in the direction of their orientation [23]. Therefore, aligned polymeric nanofibers are preferably applied in ligament [24], nerve [25] or muscle [26] regeneration with the aim to specify the direction of tissue growth. Randomly oriented nanofibers find the applications in skin [27] and cartilage [28] tissue engineering, where all directions of growth are necessary. For effective cell cultivation, however, all kinds of polymeric nanofiber scaffolds must fulfill the following criteria:

- Porosity and pore size,
- Mechanical properties (e.g., strength and resistance),
- Biocompatibility,
- Susceptibility to attachment of cultivated cells,
- Biodegradability (in case of biodegradable materials),
- External geometry,
- Surface properties (surface area, roughness and charge),
- Capability to surface modifications [21].

Because the fields of application of polymeric nanofiber-based scaffolds is extensive and each of them uses similar types of polymers, only one area, cartilage tissue replacement, will be described in detail. Other areas of tissue engineering are mentioned in Table 1, together with commonly used polymers.

Cartilage tissue engineering effectively uses polymeric nanofibrous scaffolds for cultivation of chondrocytes (CHs, cartilage-forming cells). High porosity of the scaffolds enables to locate cultivated cells in required area, which has a great influence on reduction of implant failure. Moreover, scaffolds could serve as a substrate for bonding of CHs, for which their anchorage is necessary for successful adhesion [29]. 2D or 3D scaffolds can be used for the cultivation of chondrocytes. 2D cultivation (e.g., in Petri dishes) may lead to the cell’s dedifferentiation (cells undergo a reversal of differentiation and lose specialized characteristics). Dedifferentiated cells then produce type I collagen instead of type II one, which can be advantageously used in specific applications requiring this collagen type. 3D mode of cultivation produces differentiated CHs with given phenotype and function. Thus, the advantage of this mode of cultivation is the chondrocytes proliferation support with maintaining of important cell functions [30]. Moreover, the use of scaffolds itself can practically accelerate and simplify the cartilage tissue regeneration, because they may act as a supporting system for the migration of CHs preserved in remaining tissue [31]. As the suitable materials for scaffolds, one can use natural, as well as synthetic polymers.

Natural polymers, applicable in this field, include biodegradable materials, such as silk [31], chitosan [32], hyaluronic acid [33], chondroitin sulphate [34], alginate [35], gelatin [36], cellulose [37] and bacterial cellulose [38] and arbitrary combination of all of them. Unlike natural ones, synthetic polymers for cartilage tissue replacement can be biodegradable, such as polyhydroxyethylmethacrylate (polyHEMA) [39], polylactic acid (PLLA) [40] and its copolymers with polyglycolic acid (polylactic-*co*-glycolic acid, PLGA) [41] or bioinert (e.g., polystyrene (PS) [42], polytetrafluoroethylene (PTFE) [43], polyethylene terephthalate (PET) [44] and polyethyl- (PEMA) and methylmethacrylate (PMMA) [45]). Bioactive hydroxyapatite should also be noted, which, however, does not belong to the category of organic materials [46].

From the group of natural polymers, biodegradable silk fibrous proteins excel in unique mechanical properties, especially processability, together with excellent biocompatibility, which makes silk a suitable scaffold material for cartilage tissue engineering. Wang et al. [31] studied the influence of 3D aqueous silk fibroin scaffolds on attachment, proliferation and differentiation of human chondrocytes (hCHs) in vitro. They revealed that hCHs had spherical morphology similar to their cultivation in physiological environment (in vivo), which promises their high effectivity in cartilage regeneration.

Another promising naturally occurring biodegradable material for this purpose is chitosan. Chitosan is a natural aminopolysaccharide that degrades into simple sugars, which are the precursors of physiological agents, such as glycosaminoglycans. In this manner the use of chitosan prevents foreign-body reactions and implant rejection. Griffon et al. [32] compared chondrocyte proliferation and function between chitosan and polyglycolic acid 3D scaffolds while using various pore size of chitosan sponges. They found improving CHs proliferation and metabolic activity with increasing pore size, which improved the diffusion of nutrients and cells throughout porous scaffold. Polyglycolic acid scaffolds produced CHs with the morphology closer to natural cartilage, however, with significant risk of above-mentioned material rejection. Combination of these two polymers may exhibit the benefits of both of them.

Biodegradable scaffold can effectively be made also from another natural polysaccharide, hyaluronic acid (or its form hyaluronan). It is physiologically occurring major component of extracellular matrix (ECM) in connective tissues, which provides their self-healing activity. Hyaluronic acid improves proliferation of CHs, because it is able to interact with these cells via various surface receptors with maintaining their original phenotype. Yoo et al. [33] prepared 3D hyaluronan scaffolds by gas foaming/salt leaching method. Such scaffolds were seeded by bovine articular chondrocytes and their adhesion, secretion of ECM, collagen synthesis and phenotypic characteristics were studied. Hyaluronan prepared an optimal environment for the growth of CHs and saved them from dedifferentiation process as well. In particular, collagen type II was found as a major characteristic marker protein in formed hyaline cartilage tissue. The formation of hyaline cartilage tissue produced CHs of native-like histological morphology having correctly differentiated phenotype.

Another component of animal cartilage tissue is mucopolysaccharide chondroitin sulphate, thus, it is a suitable material for the preparation of scaffolds. It can be classified to the group of glycosaminoglycans that covalently link to a protein core form proteoglycans. Due to these characteristics, chondroitin sulphate plays a physiological role in the intracellular signaling, cell recognition and communication of ECM with surface components of cells. Above that, chondroitin sulphate is able to direct cell orientation and pathfinding. Chang et al. [34] investigated 3D chondroitin sulphate porous scaffolds by particulate leaching. Because chondroitin sulphate is strongly water-soluble biodegradable polymer, it noticeably increased water-binding capacity, which contributed to the spreading, migration and growth of CHs. This phenomenon caused scaffold mimicking of the original environment.

Novel strategy, how to repair damaged cartilage, is the use of alginate. Alginate is a natural polysaccharide, widely distributed component of the cell walls of brown algae as calcium, magnesium or sodium salt of alginic acid. The most common calcium alginate is biodegradable, water-insoluble, gelatinous substance efficient in entrapment of enzymes and suitable for tissue culture. Wang et al. [35] prepared a highly organized 3D alginate scaffold for cartilage tissue engineering by microfluidic technology. This technique produced alginate bubbles, which formed hollow beads and became highly organized 3D ordered array structures. Their collection then formed a sponge-type, multiple-layer alginate scaffold with uniform pore size, high swelling ratio and porosity. Alginate scaffold exhibited no cytotoxicity to CHs, which exhibited normal cell phenotypes, proliferation and functionalization. Ongoing animal studies suggest that this scaffold provide new possibilities for cartilage tissue engineering in the near future.

Another novel material for this purpose is gelatin. Gelatin is a product of degradation of collagen, which, besides others, is the major protein component of cartilage and ECM. Unlike to collagen, gelatin has relatively low ability to trigger the immune system’s response by the production of antigens, which makes it more suitable material for cartilage tissue replacement compared to its precursor. In addition, gelatin can promote the information signals, which can improve cell adhesion, differentiation and proliferation. Lien et al. [36] prepared 3D gelatin scaffolds of various pore sizes by crosslinking, seeded them by articular CHs of Wistar rats, which were cultured in these scaffolds in vitro. They found that the pore size of the scaffold increases with increasing crosslinking temperature. While the cells in the scaffolds with the smaller pores were dedifferentiated, the larger pores better maintained their phenotype. Chondrocytes also better proliferated with larger pore size and produced higher amount of ECM in the group of scaffolds. Thus, the pore size was a key factor for cell metabolism.

The last natural polymer reported in literature as a suitable material for cartilage tissue engineering is cellulose. Cellulose is the most widespread polysaccharide in nature, which is water-soluble at short length of polymeric chains and can be biodegraded by enzymatic processes. Moreover, bioadhesive cellulose in cartilage tissue engineering is easily processed to 3D scaffolds suitable for growing functional CHs in vitro. These types of scaffolds were studied by Müller at al. [37]. They demonstrated the ability of cellulose scaffolds to significantly enhance cell adhesion, proliferation and vitality, indicating the excellent biocompatibility of this material. The distribution of the seeded CHs was homogenous and proved the development of cartilaginous tissue. Measured production of calcium and formation of the precipitated layer showed the development of microenvironment that ordinarily led to reparation in the vicinity of subchondral bone in vivo. In particular, cellulose scaffolds were able to repair not only the damaged cartilage, but also the subchondral bone, which integrate the neo-cartilage into the osseous surrounding. A special kind of cellulose is bacterial cellulose, which is produced by bacteria, such as *Gluconacetobacter xylinus* (also called *Acetobacter xylinum*). Bacterial cellulose, as biodegradable natural polymer, is a novel biomaterial with unique properties including high water holding capacity, high crystallinity, fine fiber network and high tensile strength. Svensson et al. [38] reported response of primary bovine chondrocytes on bacterial cellulose. They found that 3D cellulose scaffolds supported CHs proliferation at about 50% level and production of the collagen type II, confirming that chondrocytes maintain their differentiated form, while the porosity of the material did affect CHs viability. The scaffold supported cell ingrowth too. Moreover, bacterial cellulose did not induce significant activation of proinflammatory cytokine production during in vitro testing. For these reasons, bacterial cellulose exhibits potential for the application as scaffolds for tissue engineering of cartilage. To preclude the disadvantages of individual polymers and ensure better mimicking of natural environment, the combination of these materials can be effectively used for these purposes [75].

As natural polymers, the synthetic ones must also fulfill the conditions of biocompatibility, high porosity and cell-adherence capability for their successful applications in cartilage tissue engineering. They must act as a network on which the cells can proliferate, while preserving the shape of the injured cartilage. One of the representatives of biodegradable synthetic polymers is polyHEMA. PolyHEMA is a soft, flexible, water-absorbing polymer that forms a hydrogel in water. For the applications, polyHEMA chains are often cross-linked into 3D network by another polymer to form a copolymer. This leads to the formation of transparent material that absorbs up to 40% of water and exhibits unique mechanical properties. Its application in intraocular contact lens is well established, but such 3D network has also suitable properties as the scaffolds for cell cultivation. In this case, it induces spheroid formation of cultured cells. Bölgen et al. [39] prepared biodegradable copolymer polyHEMA–lactate–dextran (pHEMA–LLA–D)-based 3D scaffolds for cartilage tissue engineering and studied its biocompatibility with bovine articular cartilage CHs in vitro. They revealed cells’ growth on the surface and within the scaffolds, while most cells were attached to and integrated with the pores of the scaffold. The chondrocytic morphology was round to oval-shaped. Cells rapidly proliferated, secreted significant amount of ECM and were interconnected with each other by communication junctions. Due to their unique properties, such as soft, elastic nature, highly open interconnected pore structure and very rapid, controllable wettability, the pHEMA–LLA–D scaffolds are excellent candidates for cartilage tissue regeneration.

Another synthetic, but natural-based biocompatible polymer is polylactic acid. PLLA is polymer derived from lactic acid, which comes from renewable resources (fermented plant starch, such as from corn, cassava, sugarcane or sugar beet pulp). PLLA undergoes hydrolytic deesterification to lactic acid in vivo, which is a normal cellular metabolite that degrades by the Krebs’s cycle in lungs into carbon dioxide and water. The absence of peptide linkages in polymeric chains minimizes immunogenicity (ability to provoke an immune response). In particular, the biodegradation without immunogenicity is the key factor, for the use of PLLA as a scaffold for cartilage tissue engineering. Chu et al. [40] determined the suitability of porous D,D-L,L-polylactic acid as a 3D scaffold able to repair cartilage tissue into full-thickness articular cartilage using rib perichondrium. By characterizing both, the cell-polymer implant and the resulting repair tissue following implantation, the authors demonstrated that perichondrocytes are able to attach to and survive within the scaffold and that a consistent, cartilaginous-appearing repair was formed when implanted into drilled osteochondral defects in a rabbit model. Thus, PLLA-based scaffold has the potential to create viable cartilage healing. Novel approaches, however, combine polylactic acid with another biodegradable polyester, polyglycolic acid, to gain better properties and ability to mimicking of cartilage tissue. The use of PLGA copolymer enables to control of biodegradation period by the setting of various ratio of each polyester. Moran et al. [41] studied the influence of such 3D scaffolds composition on the physical properties, adhesion and growth of bovine articular CHs. They found that the compressive modulus of scaffolds increased linearly with the addition of PLLA, as well as the biodegradation period (from 5 to 45 days). Addition of PLLA then decreased cell seeding efficacy and the morphology of cells changed from flat to more round with increasing amount of polylactic acid. This study provided important information for the design of scaffolds for cartilage tissue engineering.

The group of bioinert synthetic polymers for cartilage tissue engineering represents polystyrene (PS). PS is non-biodegradable aromatic polymer that can exist in various forms. Tissue culture polystyrene (TCPS), used for cell cultivation, is specific polystyrene modified by plasma treatment. This inexpensive, disposable and transparent polymer is well known as cultivation standard. It improves the attachment and proliferation of cells and enables their connection with other cells and ECM. Intra- and extracellular communication then ensure cell apoptosis (programmed cell death), morphology, function and differentiation. Binding of specific molecules, such as proteins (e.g., collagen) and polysaccharides (heparin), on the surface of TCPS can mimic the biologic environment, from which the specific cell type is derived (for more details see paragraphs below). Sato et al. [42] investigated the potential of collagen/heparin-carrying polystyrene (HCPS) 3D scaffold for articular cartilage tissue engineering. This scaffold built an optimal extracellular environment with a high-performance extracellular ECM that enabled the aggregation of heparin-binding growth factors within the scaffold. Culture of rabbit articular CHs exhibited high cell proliferative activity, because an extracellular environment was similar to physiological one and heparan sulphate proteoglycan was generated within the HCPS scaffold.

Another synthetic polymeric material for non-absorbable scaffolds is polytetrafluorethylene. Polytetrafluoroethylene (Teflon) is a synthetic thermoplastic fluoropolymer. It may exist in the form of expanded PTFE (ePTFE), a material incorporating a fluoropolymer membrane with micropores, which are necessary for the successful scaffold applications. Since ePTFE exhibits some inappropriate properties for cell cultivation (e.g., hydrophobicity, see below), it needs to be modified before application. Springer et al. [43] prepared ePTFE 3D scaffold containing polyamide monofilaments with the aim to achieve non-absorbable scaffold material acting as a stress-absorbent network for cartilage tissue-engineered transplants. They investigated the adhesion, spreading and ECM synthesis of human and porcine CHs. Chondrocytes proliferated and spread vividly throughout scaffold, differentiated predominantly into spherical shape and produced considerable amount of ECM. Authors also identified collagen type II within the extracellular matrix, indicating no cell dedifferentiation. Observed production of fibrocartilage-like tissue when being exposed to functional stress showed the ability to balance the mechanical stress.

Polyethylene terephthalate can be also classified as a convenient synthetic bioinert material for scaffolds. It is the most common thermoplastic polyester, which is advantageously used in various application areas, because of its fiber-forming properties. Neves et al. [44] prepared polyethylene terephthalate 3D scaffold formed from PET fabric with narrow size distribution of pores by stirring-induced mixing. They studied the similarity of the response of sheep menisci fibrochondrocytes on the scaffold to physiological meniscal cartilage. Rapid cell proliferation with maximal cell densities achieved within the first seven days of cultivation, homogeneous cell distribution and the high levels of collagen and glucosaminoglycans were observed. Thus, the synthetic meniscal construct successfully integrated into a repair site in the knee that can support the quickness of healing in less than 7 days was developed.

Novel trend in synthetic bioinert polymers for scaffolds represent polyethyl- and/or polymethyl- methacrylate. These amorphous transparent polymers exhibit unique mechanical properties and low water absorption capability. Due to their biocompatibility and in vivo stability, they are used in various implants and medical devices, in which they often form nanocomposites with PS and organically modified clay as emulsifier. Vikingsson et al. [45] studied long-term mechanical stability of a composite scaffold containing a mixture of low molecular weight PEMA and PMMA and applicable in cartilage defects. They exposed the scaffold to compression cycles and found that the dry scaffold failed after a smaller number of deformation cycles than water-immersed one, which was not affected by 100,000 compressive cycles. Water immersion simulated the physiological environment after implantation. Therefore, the scaffold is able to exhibit great mechanical performance when implanted in chondral defects. This form of implantation of empty scaffold also supported the regeneration of new tissue inside the scaffold’s pores.

**Figure 1:**
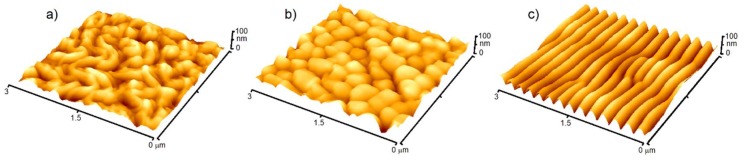
Various surface nanostructures onto polyethylene terephthalate induced by KrF excimer laser irradiation, (**a**) worm-like, (**b**) globular and (**c**) ripple structures. Measurements were carried out by atomic force microscopy.

**Figure 2:**
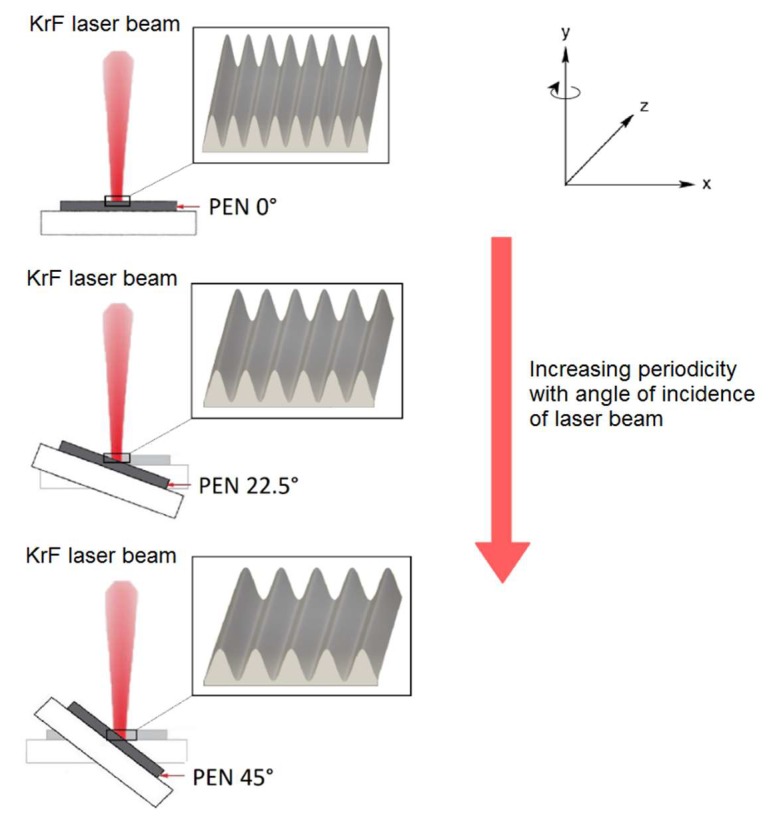
Scheme of surface nanostructuring of polyethylene naphthalate (PEN) by KrF excimer laser irradiation under incidence angles of 0, 22.5 and 45° [80].

In general, cells are sensitive to the surrounding environment, such as chemical composition and morphologic aspects of the surfaces with which they are in a contact. Polymeric scaffolds are advantageously used because of the excellent processability, biodegradability and other useful properties, however, the suitability for the applications in biologic media must be promoted in majority cases to better mimicking of physiological environment [45]. One approach, how to achieve required properties, is the modification of the polymeric surfaces. Principally, the surface of the scaffold is the primary site of interaction with surrounding cells and tissue. For this reason, scaffolds with a large and accessible surface area, appropriate roughness and hydrophilicity are favorable [76]. These parameters can be effectively increased by the exposure of polymeric surface to excimer laser beam which is able to create various forms of nanostructures on the surfaces of aromatic polymers [77]. The surface modification in water environment leads to the formation of worm-like structures, which passes to globular-like ones with increasing laser fluence (see Figure 1) [78,79]. When exposing the polymeric surface to laser beam under various angles of incidence on air, one can obtain the nanostructured, highly-ordered periodic surface structures, such as ripples (Figure 1), whose periodicity increases with increasing incidence angle (see Figure 2) [80]. The use of such structures for the cell cultivation leads to orientation of cells in direction of the nanostructures. This phenomenon can advantageously be used in applications, where structured cellular arrays with influenced cell behavior is necessary [81].

The surface, however, exhibits both topographical and chemical characteristics. Biocompatibility of polymeric scaffolds can also be improved by the addition of various compounds, which can be physically adsorbed or chemically bounded on scaffolds surface. The immobilization of proteins, saccharides or other physiological compounds forms specific binding sites onto scaffold surface and may increase polymer hydrophilicity [45]. Thus, the resulting surface scaffold properties can be selectively modified to optimize material properties for hosting cells. Cells are able to specifically recognize the binding sites and their proliferation, growth and/or differentiation can increase [76]. Another possibility is the integration of other biologically active compounds, which are not physiologically-based. This group of materials represents inorganic nanostructures, whose unique properties are described in following text.

## 3. Inorganic Nanomaterials

Inorganic nanomaterials for bioapplications are represented mainly by silica, metals, magnetic, ceramic and carbon-based materials, especially in the form of nanoparticles and nanotubes (NTs). Their applications in diverse areas of tissue engineering are summarized in Table 2 and described in following chapters. Other bioapplications of these nanomaterials, such as bioimaging [6], drug [4] and gene [82] delivery should be mentioned too. Especially nanoparticles find application as drug delivery containers, material reinforcements and agents controlling the release of bioactive molecules [4].

For the application of inorganic nanomaterials in the living organisms, one must consider all aspects which may lead to rejection of such artificial material by living body, even on the cellular level. The most important property is biocompatibility. The material in a human body must be in harmony with ambient tissues and it must not trigger unwanted processes like organs dysfunctions, intoxication or allergy. Biocompatibility of inorganic nanomaterials differs from that of “classic” bulk materials. Because of their small size, they can enter body fluids (blood, plasma, extracellular matrix), where they can interact with proteins [83]. Due to their affinity, they can be absorbed by cell organelles and affect processes such as cell breathing, DNA transcription or transport abilities of cell membrane [84]. Nanoparticles can also be combined with antibodies, which may increase their biologic effect [85].

Silica is biocompatible material with excellent chemical stability and surface properties [86,87]. Silica NPs can occur as individual objects for imaging or drug delivery or as coatings of other materials. The most known bioactive coating is tetraethyl orthosilicate. It can be combined with ammonium hydroxide and deionized water [88]. The size of silica nanoparticles can be affected by specific solution composition, especially by water to ammonium hydroxide ratio. Size and shape depend on final usages, so nanoparticles could be in the form of shells, mesoporous and bulk silica particles varying in dimensions [87,89,90]. Mesoporous silica particles have broad range of desired properties, e.g., they can absorb large amounts of biomolecules, and are able to resist to heat, pH changes, mechanical stress and hydrolysis-induced degradations. These particles are also treasured for chemical and thermal stability, simple fabrication and nontoxic nature [86,91].

Metal nanoparticles represent dynamically developing group of nanomaterials which excel in sensing, catalysts and therapeutic application, especially due to exceptional optical properties and properties resulting from inner electron states, which completely differs from those known in bulk counterparts. Within this group of nanomaterials gold nanoparticles occupy an exceptional position, owing to their excellent biocompatibility and considerably low cytotoxicity [118]. Generally, metal NPs exhibits unique antibacterial properties, well known for silver [119]. They may be synthesized in many shapes and sizes, which can easily be identified by surface plasmon resonances visible in UV-Vis spectra [120]. Despite of many favorable properties predetermining metal NPs for incorporation in vast range of advanced functional products, their toxicity [1,121], biocompatibility [119,122], bioremediation [123] and overall environmental impact must be constantly controlled during the process of their synthesis, handling and especially at the end of their lifetime [124].

Strong UV absorption, mainly exploited in cosmetics, is well known for TiO_2_ nanoparticles. They are able to preclude the penetration of sunlight into skin while remaining transparent layer [125]. Their combination with silica [126] and/or alumina [127] coatings prevent photocatalytic reactions. Compared to other substances used for UV protection, TiO_2_ nanoparticles are one of the safest for dermal exposure. However, the inhalation exposure may potentially bring a lung cancer risk, thus, standard hazard controls for TiO_2_ nanoparticles are necessary [128].

Magnetic nanoparticles comply the Coulomb’s law. They can be manipulated (guided) by an external magnetic field. They are applied in the transport and/or immobilization of magnetic nanoparticles with magnetically bound biologic substances to the targeted area. By this way, the delivery of anticancer agents to the tumor site is performed. This way of controlled drug delivery streamline the treatment while reducing the side effects [129]. Moreover, energy transfer from the external magnetic field to the magnetic NPs occurs, which leads to their heating and tumor cells are thermally destructed due to hyperthermia [130]. Hyperthermia also cause the excellent antibacterial properties of magnetic NPs by thermal destruction of bacterial cells [131]. As well, magnetic nanoparticles are used in bioimaging for the increase of the contrast in magnetic resonance [132]. In bioapplications, classic intrinsically magnetic materials, such as iron [131], but also semiconductors (magnesium oxide [133], zinc oxide [134] and selenium NPs [135]) are commonly used. In particular, magnetic properties of semiconductors can be achieved by the preparation of thin films with crystalline structure, which can be deposited by magnetron sputtering. Magnetism without d-orbital electrons is based on polarization induced by p-orbitals while the contribution of such developed defects to the resulting magnetism is different when appear on the surface or subsurface layers of the crystalline thin film [136]. MgO have this behavior associated with the spin triplet state of Mg vacancy and magnetic interaction occurs through-bond spin polarization between two Mg vacancies [137]. The existence of spin-polarized carriers in ZnO is the result of interactions between the conduction band and magnetic impurities (i.e., s-d exchange interaction in transition metal-doped ZnO) [138]. In case of Se (e.g., in organoselenium compounds), mixing of σ and π orbitals results in a large paramagnetic contribution to the resulting magnetism while chemical shift and spin–spin coupling can be observed [139].

Except of single oxide NPs (e.g., TiO_2_, MgO and ZnO) described elsewhere, the group of bioceramic nanomaterials consists of nanostructured hydroxyapatite [140], calcium-defective hydroxyapatite [141], tricalcium phosphate [142] and bioactive glass (mixed oxides SiO_2_-CaO-P_2_O_5_) [112]. Chemical composition of these materials is close to natural bone and tooth. Except the widespread applications for bone tissue replacement [143], they can also serve as anti-erosive agent in toothpastes for the prevention of dentine erosion and its remineralization support [140]. As applied in tooth implants, they excel with significant antibiofilm activity. It plays an important role in the prevention of gingivitis [144]. Another application of bioceramic nanoparticles is the controlled delivery of drugs or genes [145,146].

Bioapplicable carbon-based nanomaterials include carbon nanotubes [147], graphene [82] and graphene oxide (GO) [117]. Carbon nanotubes represent rapidly growing biologically applied nanomaterial in last decades. They can deliver drugs and genes, help in theranostics, biosensing and in enhancement of microscopy resolution [147]. Carbon atoms are bonded in sp^2^ hybridization, which gives CNTs good electrical and thermal conductivity and high mechanical and chemical stability. They can exist as (i) single nanotubes with a diameter range of 0.4–2 nm or as (ii) multiwalled CNTs with an external diameter range of 1.4–100 nm [148]. They can be conductive or nonconductive due to their specific size. The toxicology of CNTs is not still clearly known, however, some pioneering studies are warning about their harmfulness when exposed to rats [149]. Shape, length, surface charge, diameter, purity and agglomeration are the factors, which predetermine level of CNTs toxicity [150]. Alike CNTs, graphene and its modified forms are favorable materials for bioapplications for their unique properties. Graphene is a crystalline form of carbon represented by only one layer of sp^2^ hybridized orbitals tightly packaged into 2D hexagon grid. Each carbon atom has three σ-bonds and one π-bond outside the plane that can bind to adjacent atoms [151]. In addition, graphene sheets can be transformed into other conformation (e.g., GO) by chemical or physical modifications [117]. Moreover, graphene may be packaged into a form of 0D nanomaterial, such as fullerene or rolled into 1D nanotubes (1D), it may also form 3D graphite [152]. Each of these modifications has unique and tunable properties, but they also exhibit a toxicity (similarly to CNTs). The properties of all described carbon-based nanomaterial are exploited in bioimaging [6] and drug or gene delivery [82].

## 4. Opportunities of Properties Enhancement

The predominant group of scaffold materials for tissue cultivation forms polymeric nanofibers. These organic materials, however, often exhibit inappropriate properties (for detail see Chapter 2). The great opportunities for mimicking the physiological environment are gained by the incorporation of inorganic nanomaterials [153] able to improve the design and functionality of resulting material and thus, to overcome limiting factors of single material. The composite material design can be modified for the applications locally in tissues, systemically throughout the body and at the interface with tissues. Such modification requires detailed understanding of both biologic and material properties, which is prerequisite of the development of more sophisticated biomaterials that achieving specific therapeutic goals in tissue regeneration.

### 4.1. Biologic Properties

Gold nanoparticles (AuNPs) and titanium dioxide nanoparticles (TDNPs) provide high cell proliferation rates for bone and cardiac tissue regeneration. Although AuNPs itself possess superior biocompatibility, their properties may also be quite easily modified, which is especially interesting in biomedical application [154,155].

They have been reported to assist in differentiation of osteoblast precursor cells [156], having direct influence on osteoclast formation from hematopoietic cells during protective processes on mitochondrial dysfunction [95]. The size of the AuNPs, however, has crucial influence on the cell proliferation. While the particles in the range of 30–50 nm effect human adipose-derived stem cell function [156], those of 20–40 nm size influence osteoblast-like cell function [157].

AuNPs have excellent properties for replacement of bone morphogenetic proteins. They exhibit considerable effects on bone regeneration and promote repair processes in these tissues. However, high cost, unwanted inflammatory reactions and uncontrolled bone formations make AuNPs candidates, which still must be carefully developed to fit desired applications [158]. There have been some studies pointing to promotion of osteogenic differentiation, when AuNPs are seeded in gelatin. With increasing AuNPs concentration, the proliferation of osteoblasts was enhanced [159]. AuNWs may also direct stem cell differentiation without distortion of growth factors [160]. This finding is revolutionary in the view of functional organ transplantation due to minimizing the side effects of used growth factors in the body.

**Figure 3:**
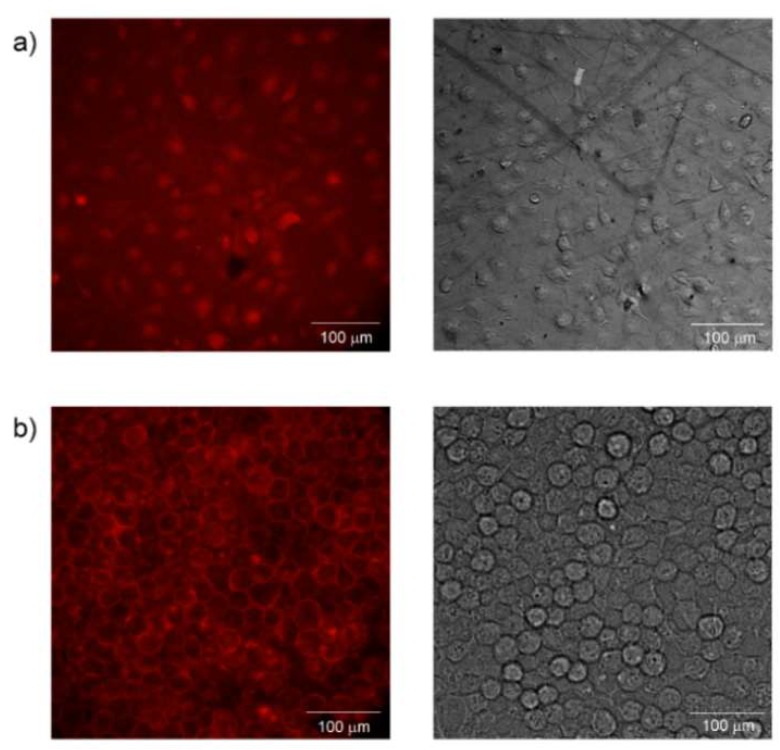
Mouse embryonic fibroblasts L929 growing on the surface of (**a**) pristine polytetrafluoroethylene and (**b**) polytetrafluoroethylene coated by Au nanolayer. Images were taken by fluorescent microscope at magnification of 40×.

Enhancement of cell proliferation of human embryonic stem cell-derived cardiomyocytes can also be accomplished by adding TiO_2_ nanoparticles to the scaffold [103]. TiO_2_ nanoparticles can be also used in 3D printing technology of polymer as polylactic-*co*-glycolic acid to significantly improve bone cell performance by matching the nanostructured roughness of bone itself. Moreover, TiO_2_ nanoparticles play an important role in preventing the harmful effects of dangerous products released during PLGA degradation, which causes the cell death [101]. Promotion of biocompatibility by the nanometal coating treatment of polymeric surface is shown in Figure 3.

### 4.2. Mechanical Properties

Inorganic nanoparticles may be used also as reinforcement improving scaffolds mechanical properties. Several studies have shown that NPs-embedded artificial scaffolds (polymeric electrospun nanofibers and hydrogels) provided superior mechanical properties for tissue engineering application. Scaffolds without nanoparticle-reinforcement did not provide these preferable properties [161]. Some nanostructured forms of TiO_2_, e.g., nanotubes, are treasured for their excellent properties as high tensile strength, lower modulus, biocompatibility and excellent thermal stability. For improving the mechanical properties of scaffolds used in skin tissue engineering, the 3D nanocomposites are usually used, which consist of type I collagen and TiO_2_ nanoparticle-coated polyvinylpyrrolidone (PVP). In this mixture, the hydrogen bonds between collagen, PVP and TiO_2_ nanoparticles play the key role in increasing of tensile strength [104]. Not only Au or TiO_2_ nanoparticles can be used for increasing of mechanical strength, but also magnetite nanoparticles (MNPs) increase the mechanical strength (e.g., MNP-seeded hydrogel microfibers with poly(N-isopropylacrylamide)) [162]. Due to rapid developments in nanotechnology, there are many other examples of mechanical properties improvement in NPs-reinforced nanocomposites, where AuNPs, AgNPs, MNPs, TiO_2_ NPs, CaCO_3_ NPs, hydroxyapatite and PVA nanofibers, play a key role.

### 4.3. Electrical Properties

Inorganic nanoparticles with electrical properties can be utilized in cardiac and neural tissue engineering [115,163]. Actually, AuNPs seem to be the most promising in this field. Shi et al. [164] reported that deposition of AuNPs into fibrous decellularized matrices improved resulting morphology of cardiac cells grown within these scaffolds. This provides better striation behavior and improved electrical coupling of proteins. Afterwards, cardiac cells started proliferation through the 3D porous structure of scaffolds which resulted in synapse formation [165]. CNTs are also used as a conductive material in neural tissue engineering [166] and in composite with polymer for improving conductivity in cardiac tissue engineering. For more about cardiac tissue engineering see below.

### 4.4. Antibacterial Properties

**Figure 4:**
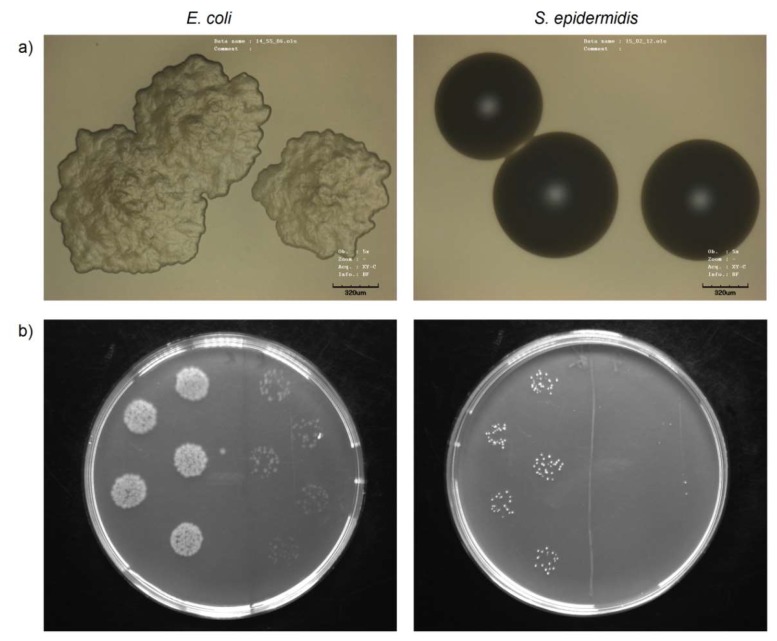
Images of bacterial strains of *E. coli* and *S. epidermidis* taken by confocal microscopy (**a**) and growing on agar plates (**b**), before (left) and after (right side of agar plate) the treatment with Ag nanoparticles.

Nanostructured forms of heavy metals, such as silver [167], gold [168], copper [169] and others [170], are able to exhibit strong antibacterial properties by the mechanism generally known as oligodynamic effect. This effect runs in low concentrations, in which the metals react with thiol or amine groups of bacterial proteins and form a covalent bond. Covalently bonded proteins then lose their function and bacterial cell perish [171]. The bioapplications of nanostructured heavy metals require also good biocompatibility, which is typically size-dependent [172]. The achievement of both, strong antibacterial activity and biocompatibility can be effectively exploited in various bioapplications of materials. Nowadays, these nanostructures are especially effective in the fight against hospital-acquired infections [119], which are commonly caused by frequently occurred environmental microorganisms, e.g., Gram-negative *E. coli* and Gram-positive *S. epidermidis* (see Figure 4).

Metal nanoparticles also combine antimicrobial effects and wound healing capabilities [173]. Presence of silver in poly(3-hydroxybutyrate-*co*-3-hydroxyvalerate) (PHBV) nanofibrous scaffolds provided the antibacterial activity, which significantly promoted cell compatibility. AgNPs can be also used in bone tissue engineering as a supplement in biocomposite scaffold. Scaffolds and bone silver-containing cements can regulate bacterial infection during the reconstruction of bone tissues and prevent inflammation [99,173]. Not only scaffolds, but also implants represent potentially attractive groups of materials in antibacterial treatment. Thin nanolayer of silver in form of nanoislands [3], nanoparticles [174] or nanowires [175] serves as a protection against infection, sepsis and malfunction of implants [173]. Composites as hydroxyapatite-AgNPs and hydroxyapatite/collagen-AgNPs scaffolds were developed as potential artificial graft materials for bone tissue engineering. In skin tissue engineering, AgNPs have been successfully applied to promote antibacterial activity of carriers. The scaffold made of collagen and AgNPs was used for burning healing, where silver particles helped to keep the congruent environment for successful curing without inflammatory reactions [100]. From the group of noble metals, Pd and Pt-based nanomaterials have been also repeatedly reported as antibacterial promoting agents [176,177]. One of the many benefits of Pt oxides is less harmful impact on the environment.

Not only the group of noble metals, but also magnetic nanoparticles have significantly effective antibacterial properties. Magnesium oxide nanoparticles and their halogen adducts with Cl and Br [133,178] consistently showed strong antibacterial properties. Results of the study showed that these nanoparticles are effective towards three groups of bacteria: *Escherichia coli*, *Bacillus megaterium* and *Bacillus subtilis*. The fascinating finding was the elimination of *E. coli* and *B. megaterium* bacteria in only 20 min. Another antibacterial agent belonging to this category, selenium nanoparticles (SNPs), are very effective in killing of bacteria by different mechanisms compared to e.g., silver ones [135]. Published results from live/dead assays implied that the SNPs killed approximately 40% of *S. aureus* after 3 h. This inhibitory effect of SNPs at early time points that SNPs may prevent *S. aureus* from forming biofilms. In addition, Iron oxide nanoparticles are effective in destroying bacteria after biofilm formation [131]. They can penetrate the biofilm (antibiotics cannot do this) and kill bacteria from the “inside”. Antibacterial effects of this group of nanoparticles, however, occur only in the presence of a magnetic field, which brings them a great application potential in tissue engineering.

## 5. Composite Organic/Inorganic Nanomaterials

In the field of tissue engineering, composite organic/inorganic materials are used as the hierarchical 3D scaffolds for cartilage, bone, neural, cardiac, skin and other tissues. In this view organic materials, represented especially by polymeric nanofibers (as described in Chapter 2), serve as a matrix for the integration of inorganic ones. Inorganic nanostructures can control adhesion and promote growth and differentiation of cultivated cells. In the following text examples of the applications of such organic/inorganic composites in the selected areas of tissue engineering are given.

### 5.1. Cartilage Tissue Engineering

As generally known, the cartilage of adults cannot be regenerated after an injury. This is caused by the absence of vascular networks and chondrocytes, which cannot be caught on a dense extracellular matrix. Thus, the healing potency of cartilage is limited. Moreover, synthesis of cartilage is very difficult due to their low proliferation. Current materials engineering, however, must react on this objective obstacle and offer the ways to enable preparation of artificial materials mimicking the function and properties of natural cartilages, suitable for implantation [179]. For synthesis of artificial cartilages, polymers are the best candidates, especially in the form of nanofibrous scaffolds (for more details see Chapter 2). The scaffolds must mimic natural cartilage and support the growth of cells, with which the integration of inorganic nanomaterials can significantly help.

Arjmandi et al. [92] integrated silica NPs into alginate-polyacrylamide (ALG-PAAm) polymeric network (IPN) and investigated its potential to replace cartilage tissue. Resulting nanocomposite was studied to obtain its mechanical and tribological characteristics in dependence on SiNPs concentration. Authors determined ultralow coefficient of friction, high wear-resistance and tunable elastic and viscoelastic behaviors. These characteristics were dependent on the interfacial binding between SiNPs and ALG-PAAm matrix, enabling effective transfer of mechanical stress between these two components. The most promising candidate for cartilage tissues replacement was the composite containing 4% concentration of SiNPs, which exhibited the best mechanical properties.

Another example of suitable inorganic materials are magnetic nanoparticles. Zhang et al. [105] prepared composite scaffold for cartilage tissue engineering from type II collagen, hyaluronic acid and polyethylene glycol, incorporated by magnetic NPs. While exposed to external magnetic field, the scaffold showed a reaction but maintain its structural integrity. Thus, remote magnetic direction can deliver the material to repaired tissue by physiological fluids. As well, the adhesion density was increased and cells exhibited normal and consistent morphology. Magnetic NPs may be then normally metabolized, in other words may be broken down by lysosome and excreted through exocytosis. This material closely mimics the microenvironment of extracellular matrix of cartilage tissue.

Last but not least, bioactive ceramic nanoparticles find the applications in cartilage reparation. Kay et al. [110] investigated novel composite polylactic-*co*-glycolic acid scaffold doped by nanophase ceramic for chondrocyte adhesion in vitro. Nanostructured material was prepared by chemical treatment of microstructured PLGA. Compared with the conventional surfaces with micro-roughnesses, their results provided the first evidence of increased CHs adhesion on PLGA surfaces with nano-roughness. In particular, the study provided evidence that nanostructured PLGA/ceramic composites are able to simulate the surface and/or chemical properties of physiological cartilage in nanoscale manner.

### 5.2. Bone Tissue Engineering

Due to increasing life expectancy and related development of civilization diseases there is increasing proportion of people suffering from trauma, tumors or other diseases related to bones. To cure those diseases and especially in bone repair, physicians often use autografts, allografts and xenografts [180,181]. However, these treatments are not effective enough for bone reconstruction.

The way to success in healing is application of suitably chosen scaffold. The scaffold must support the regeneration without complication. Its morphology and surface chemistry directly affect cell attachment, proliferation and differentiation [182]. In bone tissue engineering, the scaffold must supply and keep mechanical strength. To achieve these properties, preparation of material with the porous network should be the best way. The porous structure promotes attachment of cells and subsequent gripping of such artificial material in the bone [183].

Typically, the bone structure contains organic and inorganic components. The organic extracellular matrix is usually strengthened by inorganic calcium phosphate nanoparticles and hydroxyapatite crystals. Tricalcium phosphate is important for rigidity. Collagen type I and glycosaminoglycans are part of the organic phase, which makes bone tough. For bone structure mimicking, inorganic nanomaterials are the most commonly used entities. Nanohydroxyapatite, calcium silicate nanostructures, carbon nanotubes or inorganic nanofibers can be easily formed into the necessary structure. As a replacement of type I collagen, polymers are a good choice. Polymers are used usually as nanofibers. However, their mechanical strength and biocompatibility are, lower compared to calcium phosphate ceramics [12].

Li et al. [111] studied porous scaffolds from nanohydroxyapatite, collagen and poly-L-lactic acid, which were reinforced by chitin fibers. Cells caught on pores could grow and proliferate. The new bone was built in 15 weeks after the operation. The porosity of scaffold is very important for ingrowing of cells. In the study of Wei et al. [21] the influence of nano-scaled hydroxyapatite ratio on scaffolds porosity was studied. The porosity decreases with higher addition of nanohydroxyapatite. The lowest porosity was at least 89%, which is adequate for cells ingrowth.

Calcium phosphate and calcium silicate exhibit excellent biocompatibility and bioactivity. These materials are grafted as bone substitutes, where they promote a creation of new bone [184]. Recently, cement scaffolds were prepared containing calcium silicate and calcium phosphate. Sequential testing showed that scaffold containing calcium phosphate had a hierarchically porous structure for improvement of bone tissue regeneration [185]. On the other hand, calcium phosphate provided retention of secondary structure and promoted bioactivity of such constructed scaffold. In the study of Zhang et al. [186], calcium phosphate/calcium silicate bone cement with porous structure affected significantly bone reformation. This study pointed out to higher osteogenic differentiation in vitro and significant supporting of formation of the ectopic bone and regeneration of femur cavity defect in a rabbit model. Biphasic calcium phosphate increased surface area and promoted proteins formation, including bone-inducing proteins [187]. Among biogenic materials suitable for bone tissue engineering, glass nanotubes are also excellent candidates due to promotion of cell differentiation and osteogenesis [188]. They enable ions delivering and stimulate the secretion of angiogenic growth factors.

**Figure 5:**
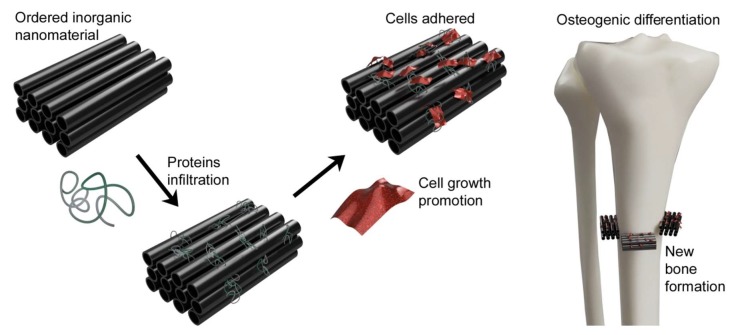
Scheme of carbon nanotubes absorbing specific protein, promoting bone formation.

Carbon-based nanomaterials occupy an important place in tissue engineering (Figure 5). The most utilized form of carbon is graphene and its derivatives. Of particular interest is graphene chemical structure. It is a monolayer of carbon atoms packed into a 2D honeycomb lattice. This structure is a base for other graphitic materials. Graphene and its derivatives possess a large surface area, high mechanical strength, intrinsic mobility, electrical conductivity and unparalleled thermal conductivity. Graphene is used as a reinforcement in a hydrogel, films fibers and other types of scaffolds contributing to their higher mechanical strength and stiffness [82]. Typical GO/hydroxyapatite hydrogels show high mechanical properties, high electrical conductivity and primarily good compatibility [151].

### 5.3. Neural Tissue Engineering

Human nervous system forms complicated network of mutually interconnected neural cells. One can distinguish two main parts of this system (i) the central nervous system (CNS, brain and spinal cord) and (ii) the peripheral nervous system (PNS, cranial and spinal nerves with ganglia). Diseases of nervous tissue are very dangerous because of a natural property of nervous system cells, i.e., their zero possibility of healing. Parkinson’s disease, Alzheimer disease, stroke, brain tumor, traumatic brain injury, infections (e.g., meningitis) are the most common diseases affecting neural tissue. The regeneration of neural tissue makes problem in tissue injury healing. The PNS shows some measure of generation property, but just in case of specific injuries. If the injury is larger and devastating, it must be surgically treated most often by limb transplantation. However, the number of donors is insufficient and it many cases is used to be even impossible to find suitable donor. Compared to PNS, the CNS injuries cannot be treated in such easy way. Neural cells network in spinal cord are complex and very specific and current science still cannot prepare artificial structure mimicking complex function of those cells. In the last few decades, however, nanotechnologies provide new materials suitable for cell migration and proliferation on the injury sites. For healing of the injury site, the variety of stem cells can be used [189].

**Figure 6:**
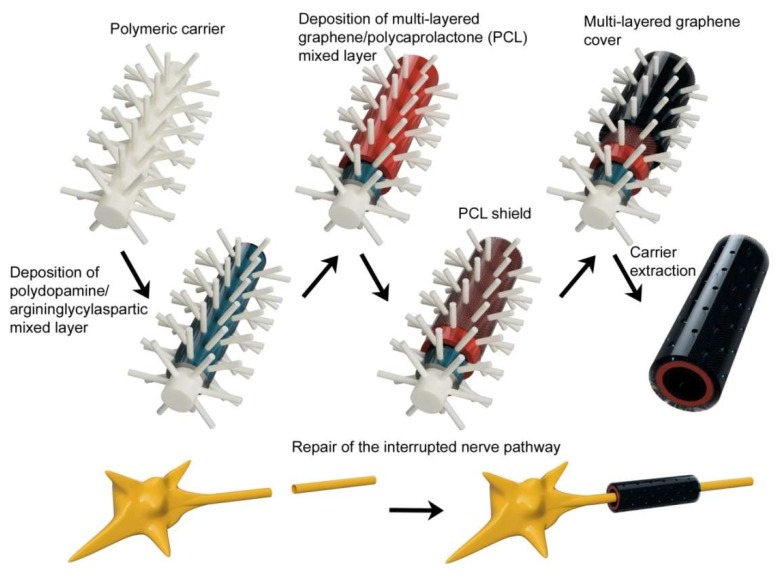
Scheme of replacement of missing part of the neuron with graphene/PCL composite using layer by layer casting method.

For neural tissue healing, carbon nanotubes are beneficially used (Figure 6). Single-walled carbon nanotubes have excellent electrical conductivity, which makes possible to support and control stem cells differentiation toward neural lineages and promote the signal transmission between neurons. The carbon nanotubes are also highly biocompatible for neural regeneration [115]. They can be used in combination with laminin for creating a thin film for cells supporting and proliferation in the synaptic connection [190]. For treating of injuries of nervous system not only carbon nanotubes, but also PLLA, acrylic acid and collagen have been reported. Nanostructured PLLA scaffolds make possible the expansion of neural stem cells [191,192,193] and present collagen can mimic the natural neuronal extracellular matrix. Combination of collagen modified with methyl methacrylate and acrylic acid in form of nanofibers provide a good environment for cells proliferation and differentiation [194], too.

### 5.4. Cardiac Tissue Engineering

**Figure 7:**
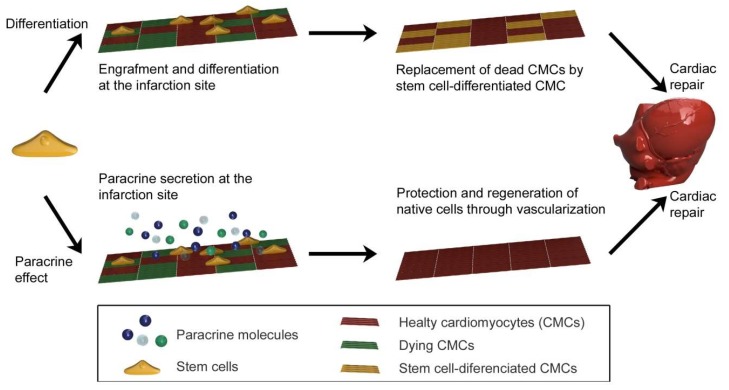
The therapeutic mechanism of dead cells replacement in cardiac tissue by stem cells.

The cardiovascular diseases represent the greatest threat for today’s mankind. In the vast majority of cases, when diagnosed at humans over 60′s, they are also the cause of death [195]. Almost 30 percent of people die on myocardial infarction. Thrombolytic treatment or surgical coronary artery bypass are most widespread used treatments of myocardial infarction. Such treatment, however, only supports tissue regeneration. Myocardial infarction is caused by reduces blood supply to the heart, the cells without blood die, the tissue is damaged, which leads up to heart failure. So, the dead cells must be replaced by new functional ones. As the new cells, stem cells (mesenchymal, embryonic, induced pluripotent and cardiac) are often used [196], which replace the cardiomyocytes (CMCs), stops cell dying and induce angiogenesis at the infarcted region (Figure 7).

Nanobiomaterials represent suitable matter for transportation of cells, proteins and genes for cardiac repair [197]. Electrical, chemical or unique topographical characteristics are welcome for creating of synergistic effects. Different combinations provide a lot of options for myocardial infarction treating [163]. The function of the heart is possible due to cardiomyocytes, which are electrically conductive. They receive the impulses from neurons, thereby making possible the right function of heart and blood circulation. Unfortunately, most electrochemical therapies are unsuitable because they may cause arrhythmias, which is uncoordinated contraction and stir conducting.

To achieve the right electrical conductivity of the heart tissue during and after the treatment, conductive nanobiomaterial-integrated therapeutics were developed with the same electrically conductive properties as cardiac tissue. The electrical conductivity is most commonly tested on carbon- and gold-based nanomaterials. The cardiomyocytes [116] and cardiac progenitor cells [198] showed good adhesion and proliferation on the polymer scaffold with carbon nanotubes or nanofibers (CNFs). The conductive nanomaterials promote expression of Connexin 43, a gene with an important role in heart development and repair. The mechanism of Connexin 43 upregulation is not yet completely clear. The understanding of therapy by conductive nanomaterials and the beating behavior of the cells on a scaffold has been briefly explained by Martins et al. [199] and Kharaziha et al. [200], which investigated the electrophysiological behavior of cardiomyocytes seeded on a chitosan/CNFs matrix or poly(glycerol sebacate)/gelatin/CNTs scaffolds, respectively. The cardiac cells have shown a synchronous beating behavior in contact with conductive nanomaterials. Increasing of expression of cardiac-specific genes, cardiac muscle contraction and electrical coupling has occurred too. Therefore, one can conclude, that the conductive materials provide the electrical coupling between cells and Connexin 43 and thus promote synchronous beating of the cardiac tissue. To improve the effectiveness of therapy, graphene NPs are also utilized. Hydrogel scaffold integrated with AuNPs [97] or AuNWs [19] increases the Connexin 43 expression by cardiomyocytes compared to CMCs cultured in hydrogel without those nanomaterials.

### 5.5. Skin Tissue Engineering

Despite that skin tissue represents an insignificant part of total body weight, skin injuries, such as burns [201] and diabetic ulcers [202], lead to the serious risks. Therefore, skin tissue engineering plays an irreplaceable role in the treatment of chronic skin wounds. The application of skin tissue replacement must be easily accomplished to rapid location and cover the wound. Then the skin cells must strongly adhere. For this reason, artificial skin tissue replacements must exhibit appropriate physical and mechanical properties to mimic physiological skin and be nonantigenic. Such prepared replacement then assimilates into the body while regenerating the new skin tissue [203]. Nowadays, skin tissue engineering develops and designs novel polymeric-based scaffolds containing inorganic nanoparticles with enhanced mechanical [104] and added antibacterial [100] properties.

Li et al. [104] developed nanocomposite polymer/TiO_2_ scaffold with enhanced stability as a potential substrate for skin tissue cultivation. Their 3D scaffold was prepared by freeze drying of the mixture of type I collagen and polyvinyl pyrrolidone (PVP)-coated TiO_2_ nanoparticles. This method generated highly porous scaffold without the use of cross-linkers or toxic reagents. The swellability, mechanical properties and hydrolytic degradation of the nanocomposite scaffolds were the key determined parameters. Authors refers that collagen, PVP and TiO_2_ were bonded by four hydrogen bonds, which increased the scaffold stability. The stability was dependent on the amount of the added PVP. The complex composition containing TiO_2_ NPs increased resulting tensile strength of the scaffold, which was four times higher than that based on simple collagen.

**Figure 8:**
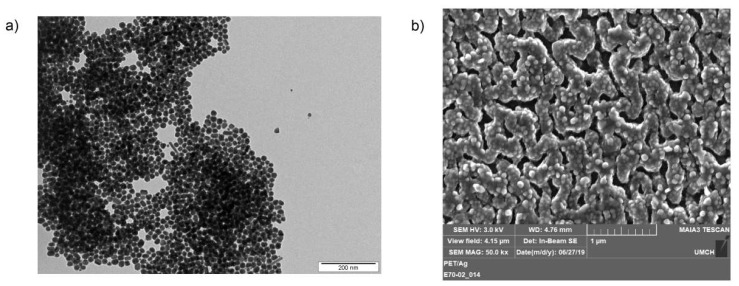
Images of electrochemically synthetized AgNPs (**a**) and AgNPs immobilized onto polyethyleneterephthalate surface by KrF excimer laser (**b**), measured by transmission and scanning electron microscopy, respectively.

Recent research predominantly emphasizes addition of antibacterial effects to polymeric scaffolds with the aim to preclude the injured skin inflammation. This capability is well known for silver nanoparticles (Figure 8a). They, incorporated into polymeric matrix (Figure 8b), can increase the healing ability of the material [20,78]. Patrascu et al. [100] studied homogenous and heterogeneous scaffold materials containing silver nanoparticles for their antiseptic behavior. Polymeric matrices were based on collagen. Silver NPs were synthetized by chemical reaction to obtain material with homogenous distribution of AgNPs and plasma sputtering for heterogenous one. In vitro assays revealed significant antibacterial activity against *Escherichia coli* at low concentrations of AgNPs (10 ppm). Thus, they developed strong antiseptic materials with the potential to repair skin injuries, especially caused by burns or cancer. For the healing of already running infection, Ag-rich side of the material must be in the contact with treated tissue. For the protective role against potential infection, both homogenous and heterogenous materials are suitable, because the contact of Ag-rich side with the skin is not necessary.

## 6. Challenges and Future Perspectives

Nanomaterials can be used in wide range of bioapplications. This review focuses on nanomaterials applicable in tissue engineering. In addition to these applications, nanomaterials open up new ways of bioimaging as well as drug or gene targeted delivery. In all bioapplications, the nanomaterials provide advantageous properties, such as enhanced cell adhesion, proliferation and growth in diverse areas of tissue engineering. Especially, they significantly promote cartilage, bone, neural, cardiac and skin tissue rebuilding by direct replacement of damaged portions or whole tissues. These fields of application are specific by the sensitivity of regenerated tissue to artificial materials and subsequent possibility of immune response. Possible risks and obstacles may be overcome by excellent biocompatibility of inorganic nanomaterials in the forms of single nanoparticles or nanotubes and their incorporation into organic (polymeric nanofibrous) scaffolds. However, there is another quite worrying point: the long-term bioaccumulation of nanoparticles in the human body can cause the diseases of unknown etiologies resulting from their toxicity and leading to harmful effects on reproductive organs, fetal development disorders or cancer. Sometimes, we cannot control each property or predict the behavior of cells. Therefore, despite the undoubted benefits that nanomaterials may bring to the society, treatments with these man-made materials possess a health risks that must be minimized by precautionary principles whenever these materials are developed, tested and then clinically applied.

## Figures and Tables

**Table 1:** Selected areas of tissue engineering together with the cultivated cell types and examples of polymers applicable as organic nanofibrous scaffolds for cell cultivation.

Site of Application	Cell Culture	Polymeric Scaffold
Bone	Osteoblasts, Mesenchymal stem cells	Polylactic acid [47], Polylactic-*co*-glycolic acid [48], Hyaluronic acid [49], Polyvinyl alcohol [50]
Nerves	Astrocytes, Schwan cells, Neural stem cells	Polylactic acid [51], Polylactic-*co*-glycolic acid [52], Chitosan [53], Gelatin [54]
Heart valves and arteries	Cardiomyocytes, Vascular fibroblasts, Vascular endothelial cells	Polytetrafluoroethylene [55], Polylactic-*co*-glycolic acid [56], Polycaprolactone [57], Polyhydroxy butyrate [58]
Pancreas	Islets of Langerhans culture	Polyglycolic acid [59], Chitosan [60], Gelatin [61], Polyvinyl alcohol [62]
Urinary bladder	Urothelial cells, Smooth muscle cells	Polylactic-*co*-glycolic acid [63], Hyaluronic acid [64], Polycaprolactone [65], Polyurethane [66]
Corneas	Corneal epithelial cells	Hyaluronic acid [67], Chitosan [68], Polyvinyl alcohol [69], Polyethylene glycol [70]
Skin	Keratinocytes, Dermal fibroblasts, Dermal endothelial cells	Polystyrene [71], Polylactic-*co*-glycolic acid [72], Chitosan [73], Gelatin [74]

**Table 2:** Overview of tissue engineering applications of inorganic nanomaterials and their main advantages.

Nanostructure type	Advantage	RegeneratedTissue
Silica NPs	Excellent biocompatibility, Uniform morphology, Chemical stability	Cartilage [92], Bone [93]
Gold NPs	Excellent biocompatibility, Low cytotoxicity, Easy functionalization	Cartilage [94], Bone [95], Neural [96] Cardiac [97], Skin [98]
Silver NPs	Antibacterial properties, Easy synthesis (various sizes and shapes) Low cost	Bone [99], Skin [100]
Titanium dioxide NPs and NTs	Excellent biocompatibility, Bioactivity, Unique mechanical, thermal and electrical properties	Cartilage [16], Bone [101], Neural [102] Cardiac [103], Skin [104]
Magnetic NPs	Long-term efficacy, Non-invasive application, Superparamagnetic properties	Cartilage [105], Bone [106], Neural [107], Cardiac [108], Corneal [109]
Ceramic NPs	Bioactivity, Biodegradability, Unique mechanical properties	Cartilage [110], Bone [111], Cardiac [112]
Carbon NTs, Graphene and its oxide	Double-sided functionality, Unique mechanical, thermal and electrical properties	Cartilage [113], Bone [114], Neural [115], Cardiac [116], Pancreatic [117]
